# Cuproplasia characterization in colon cancer assists to predict prognosis and immunotherapeutic response

**DOI:** 10.3389/fonc.2023.1061084

**Published:** 2023-03-16

**Authors:** Bomiao Zhang, Yien Li, Liqiang Song, Hua Xi, Shaoke Wang, Chenfeng Yu, Binbin Cui

**Affiliations:** Department of Colorectal Surgical Oncology, Harbin Medical University Cancer Hospital, Harbin, China

**Keywords:** copper, colon cancer, immunotherapy, microsatellite instability, immune infiltration

## Abstract

**Introduction:**

Colon cancer is the 3^rd^ most prevalent cancer worldwide, with more than 900,000 deaths annually. Chemotherapy, targeted treatment, and immunotherapeutic treatment are the three cornerstones of colon cancer treatment; however, the occurrence of immune therapy resistance is the most pressing problem to solve. Copper is a mineral nutrient that is both beneficial and potentially toxic to cells and is increasingly implicated in cell proliferation and death pathways. Cuproplasia is characterized by copper-dependent cell growth and proliferation. This term encompasses both neoplasia and hyperplasia and describes the primary and secondary effects of copper. The connection between copper and cancer has been noted for decades. However, the relationship between cuproplasia and colon cancer prognosis remains unclear.

**Method:**

In this study, we applied bioinformatics approaches including WGCNA, GSEA and etc. to delineate cuproplasia characterization of colon cancer, set up a robust Cu_riskScore model based on cuproplasia-relevant genes and found its relevant biological processes use qRT-pCR to validate our results on our cohort.

**Result:**

The Cu_riskScore is found to be relevant to Stage and MSI-H subtype, and some biological processes including MYOGENESIS and MYC TARGETS. The Cu_riskScore high and low groups also showed different immune infiltration pattern and genomic traits. Finally, the result of our cohort showed the Cu_riskScore gene RNF113A has a marked effect in predicting immunotherapy response.

**Discussion:**

In conclusion, we identified a cuproplasia-related gene expression signature consisting of six genes and studied the landscape of the clinical and biological characterization of this model in Colon Cancer. Furthermore, the Cu_riskScore was demonstrated to be a robust prognostic indicator and predictive factor for the benefits of immunotherapy.

## Introduction

Colon cancer has become the 3rd common cancer, with a mortality rate of 900 thousand in 2020, and new cases reached 1,931,590 in 2021, leading to a heavy burden on the medical industry (https://gco.iarc.fr/). Basic treatment includes chemotherapy, surgery, and immunotherapy. Immunotherapy is most effective in microsatellite instability high (MSI-H), while those with microsatellite stability (MSS) can hardly benefit from this treatment (1). Therefore, a more precise classification of sensitive patients is extremely important.

Copper is a mineral nutrient in the human body that is highly involved in cell proliferation and death pathways. The inherent oxidation-reduction (redox) property of copper makes it both beneficial and potentially toxic to cells. Recent research on the view of copper has exploited copper-dependent disease vulnerabilities, particularly in cancer (2). In particular, cuproplasia, defined as copper-dependent cell growth and proliferation, is a newly recognized form of regulated copper-dependent cell proliferation (3). This term encompasses both neoplasia and hyperplasia, describes both primary and secondary effects of copper *via* signaling pathways, and includes enzymatic and non-enzymatic copper-modulated activities. Cuproplasia can be targeted: copper signaling can be repressed by copper-selective chelators (3) or activated with metal ionophores that elevate copper levels or spatially and temporally redistribute copper stores across cellular and subcellular pools (3).

Indeed, connections between copper and cancer have been noted for more than a century, with numerous observations pointing to a requirement for higher levels of copper in tumors than in healthy tissues (2). Because of the requirement for copper as a cofactor of mitochondrial cytochrome c oxidase, which is essential to meet the energy demands of rapidly proliferating cells, Cancer cells are just the kind of quick-dividing cells consuming huge amounts of energy (4). Elevated copper concentrations have been reported in the tumors or serum of animal models and patients with many types of cancers, including breast (5, 6), lung (7, 8), gastrointestinal (9), prostate (10), and gallbladder (11) cancers. In addition, mitochondrial function and copper pathways, such as the ATOX–ATP7A–LOX pathway, can also induce metastatic expansion (12). In addition, Cuproplaisa also promotes angiogenesis, including vascular endothelial growth factor (VEGF), fibroblast growth factor 2 (FGF2), tumor necrosis factor (TNF), and interleukin-1 (IL-1). However, the cuproplasia traits and their predictive value in colon cancer remain unclear, and we thus attempted to investigate the characterization of uproplasia in Colon cancer.

## Materials and methods

### Colon cancer data collection and processing

Colon cancer samples with adequate clinical information were obtained from The Cancer Genome Atlas (TCGA) database and Gene Expression Omnibus (GEO). For the TCGA cohort (448 colon cancer samples), RNA-seq data and corresponding clinical information were extracted from TCGA database (http://cancergenome.nih.gov/) and then transformed into transcripts per kilobase million (TPM). In our study, two GEO microarray cohorts were used, and the expression and survival data were retrieved from the GEO database (https://www.ncbi.nlm.nih.gov/geo/) with background adjustment and normalized using the RMA algorithm. Before further analysis, all gene expression data were log2 transformed and quantile-normalized using the normalized between arrays technique in the R package limma 3.46.0. We eliminated batch effects from the analysis when using merged gene expression data from different datasets *via* the R package sva 3.36.0.

### WGCNA identification of cuproplasia-related genes

The Cu_score of the cuproplasia-related gene set was quantified for each colon cancer sample using the GSVA algorithm in the R package GSVA 1.36.2 (13). Weighted Correlation Network Analysis is a systems biology method for identifying correlation patterns among genes across microarray samples. WGCNA was performed using the WGCNA package in R 3.6.1. Gene significance was used to determine the correlation between individual genes and Cu_score, whereas module membership represented the relationship between module eigengenes and gene expression profiles. To ensure a scale-free topology network, a power of β = 4 and a scale-free R2 = 0.9 were set as soft threshold parameters. Following the retrieval of five modules, the turquoise module with the most solid relationship was selected for further analysis.

### Construction and validation of the Cu_riskScore

The TCGA-COAD dataset was subjected to univariate Cox regression analysis to identify genes associated with prognosis, with a p-value of < 0.01. To obtain a quantitative description of the survival risk of each patient, Lasso regression analysis was used to calculate the Cu_riskScore of patients using the R package glmnet 4.1.3, and the dependent variable of Lasso regression was patient survival days.

### Gene set enrichment analysis

The GSEA algorithm assessed the enriched biological processes between the different groups. The data in TCGA were first transformed in preparation for linear Modelling using voom in the R package limma 3.46.0. Differentially expressed genes between the two groups were calculated using the R package limma 3.46.0. Subsequently, they were pre-ranked by log2 fold-change and delivered to the R package clusterProfiler 3.18.1 for GSEA analysis. Results with an adjusted p-value < 0.05 were considered statistically significant.

### Differences in MSI and immunotherapy sensitivity between the high and low rating groups

Data on immunotherapy sensitivity of TCGA-COAD patients were extracted from The Cancer Immunome Atlas database (https://tcia.at/patients). The database can query data on gene expression of specific immune-related gene sets, cell composition of immune infiltrates, and tumor heterogeneity. According to the sensitivity scores of TCGA-COAD patients to PD-L1 and CTLA4 inhibitors in the TCIA database, the differences in sensitivity to immunotherapy between the groups with high and low Cu Cu_riskScore were analyzed.

### Annotation of tumor microenvironment cell infiltration

To examine the immune cell-infiltrating microenvironment, we quantified the enrichment levels of 64 immune signatures using the xCell algorithm (xCell: digitally portraying the tissue cellular heterogeneity landscape). We performed more thorough investigations using algorithms such as CIBERSORTx (14), ssGSEA, quanTIseq, TIMER, and MCPcell in the R package immunedeconv version 2.0.4.

### Statistical analysis

We used independent t-tests and Mann–Whitney U tests to determine statistical significance when comparing two groups with normally and non-normally distributed variables, respectively. One-way analysis of variance (ANOVA) and Kruskal-Wallis tests were used to compare differences between more than two groups (15). Spearman and distance correlational analyses were performed using R package Hmisc 4.4.1. Objects with a coefficient greater than 0.5 were considered strongly correlated (16). Cox regression analyses were performed to identify prognostic factors. The overall survival (OS) and Cu_riskScore were determined using the R package survival, and cutoff values were determined before generating all survivorship curves with the R package survminer. All heatmaps were plotted using the R package Complex heatmap 2.4.3. Data comparisons were performed using R package ggplot2. All statistical analyses were two-sided and were performed using R software. Statistical significance was defined as a p-value less than 0.05.

### Quantitative real-time reverse transcription polymerase chain reaction

From October 17, 2022, to January 19, 2023, 32 MSI patients with colorectal liver metastases receiving immunotherapy were recruited in the Colorectal Department of Harbin Medical University Cancer Hospital. Patients were required to have a metastatic size >2 cm and <5 cm before treatment and younger than 70 years. In total, 16 patients with response and 16 with non-response were enrolled. COAD tissue was obtained by core needle biopsy before the first cycle of immunotherapy. RNA was extracted using a RNeasy kit (Qiagen Sciences, Hilden, Germany), diluted using nuclease-free water, and electrophoresed on a denaturing formaldehyde agarose gel to visualize rRNA and ensure overall sample quality. RNA concentrations and purity were detected on an ultraviolet spectrophotometer (Harbin Medical University, Harbin, China). cDNA was obtained using a PrimeScript II 1st-strand cDNA synthesis kit (TaKaRa, Dalian, China). qPCR was performed using a LightCycler 480 real-time PCR machine (Roche) with SYBR. Glycer aldehyde-3-phosphate dehydrogenase (GAPDH) was used as the reference gene, and the relative gene expression was quantified using the cycle threshold (DDCT) method. This study was approved by the ethics committee of Harbin Medical University, and all patients provided a written informed consent.

## Result

### Detecting the most valuable cuproplasia-related genes using WGCNA

Cuproplasia requires the involvement of many factors (3), including ceruloplasmin as the predominant protein carrier for exchanging copper CTR1 (also known as SLC31A1) and related ion transporters for cellular copper uptake, cytoplasmic metallochaperones (ATOX1), and cytoplasmic–mitochondrial metallochaperones (CCS, SCO1, SCO2, COX11, and COX17) for targeted insertion of copper into metalloenzymes and the copper-dependent ATPases ATP7A and ATP7B, which embrace both copper export and metallochaperone functions. Metallothionein 1 (MT1) and MT2 are isoforms of thiol-rich proteins capable of binding to copper ions. Together, these proteins maintain intracellular copper homeostasis and the function of copper-dependent enzymes (**Supplementary Table S1**).

**Figure 1:**
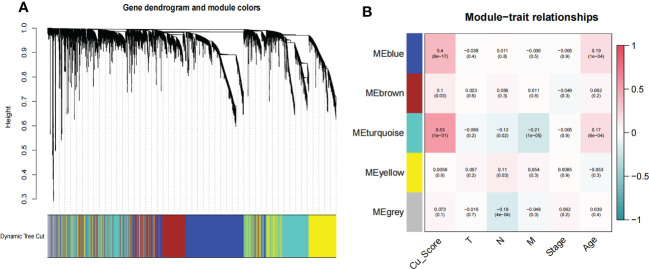
WGCNA for the uproplasia-related genes. **(A)** Cluster dendrogram generating gene modules. **(B)** Correlation analysis of modules and Cu_score, and other clinical information.

To comprehensively delineate the cuproplasia characterization, we applied GSVA analysis to score each sample in TCGA based on cuproplasia genes and defined the score as Cu_score (**Supplementary Table S2**). We used WGCNA to detect the most Cu_score-related genes (**Figure 1A**). For the scale-free network construction, the scale-free topology fit index was set to 0.9 for scale-free network construction, and correspondingly, the best power value was 4. Five modules were identified using a clustering dendrogram (**Figure 1B**). The correlation coefficient between the turquoise module and Cu_score was 0.53 (**Figure 1B**), suggesting that the turquoise module is selectively expressed in samples with a high degree of hyperplasia. In addition, this module had a strong negative correlation with the M stage of COAD, indicating the significance of the turquoise module in preventing metastasis. There were 2581 genes in the turquoise module (**Supplementary Table S3**).

### The establishment of a cuproplasia-related model

To determine the potential value of turquoise module genes, we first performed univariate Cox regression in the TCGA dataset to filter out six survival-related genes with log-rank p < 0.01(“RNF113A”,”STC2”,”POLR2J”,”PROSER2”,”ANKS4B”,”LINC01003”). To create a more interpretable model and improve the prediction accuracy, LASSO regression was used to force the sum of the absolute values of the regression coefficients to be less than a fixed value. These six genes were determined to score all samples using the following formula:0.8203*RNF113A+ 0.4026*STC2 + 0.4227*POLR2J + 0.5278*PROSER2 + -0.7549*ANKS4B + 0.0795*LINC01003. The score was designated as Cu_riskScore, and the six genes were designated as Cu-related.

RNF113A encodes a protein that contains one zinc finger domain and one ring-type zinc finger domain. The latter has been identified in various tumor suppressors, DNA repair genes, and cytokine receptor-associated molecules. STC2 encodes a secreted homodimeric glycoprotein, the C-terminus of which has been proven to interact with metal ions, and we speculate that it may regulate cuproplasia. POLR2J has been associated with immunodeficiency. However, the functions of PROSER2 and ANKS4B have not yet been well studied. Finally, LINC01003 was found to correlate with Hypophosphatasia, and from the coefficient of each RNA, we know that LINC01003 plays an auxiliary predictive role.

**Figure 2:**
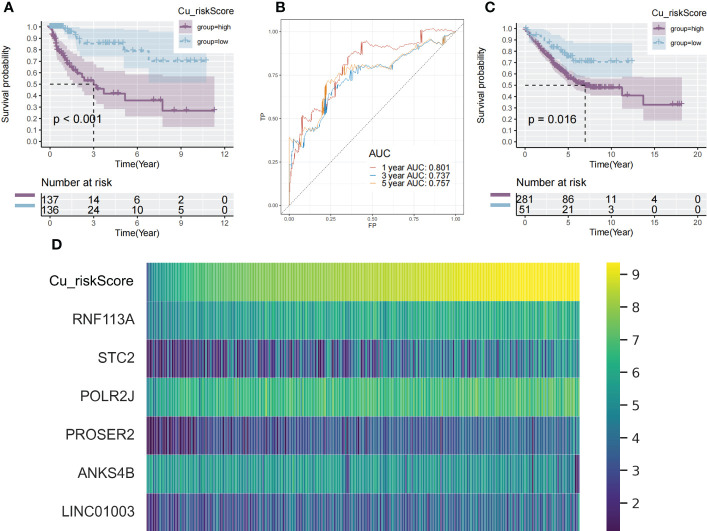
Identification of cuproplasia signature in TCGA and GEO. **(A)** The Kaplan-Meier survival analysis of the Cu_riskScore high and the Cu_riskScore low groups in the TCGA dataset, respectively. **(B)** ROC curve evaluating the value of Cu_riskScore in predicting the patient's 1-, 3-, and 5-year survival status. **(C)** The Kaplan-Meier survival analysis of the Cu_riskScore high and the Cu_riskScore low groups in the GEO dataset, respectively. **(D)** Heatmap showing the expression of 6 Cu_riskScore composing genes with increasing Cu_riskScore.

Using a median cutoff, patients in the training group were stratified into low- and high-risk groups, and Kaplan–Meier (KM) plots were generated, as illustrated in **Figure 2A**. A more specific finding was that those in the group with a reduced Cu_riskScore had significantly improved survival results with p<0.01. To further examine the efficacy of the Cu_riskScore, receiver operating characteristic (ROC) analyses were performed, yielding 1-, 3-, and 5-year Area Under the Curve (AUC) values of 0.801, 0.737, and 0.757, respectively (**Figure 2B**). The performance of the Cu_riskScore also exhibited a good predictive value in the GEO validation sets (**Figure 2C**). We then validated that the riskScore is an independent prognostic factor, by performing multivariable COX regression, including our riskScore, T, N, M grading, clinical stage, and age (**Figure S1A**). Moreover, there was no significant difference in chemotherapy, molecular drugs, and immunotherapy between riskScore high and low groups (**Figures S1B–D**). In addition, we attempted to compare our riskScore model with other existing signatures. Three signatures that were all based on the COAD data in TCGA database and all followed the same normalization workflow were collected (17–19). The formulas for the three models are listed (**Supplementary Table S4**). The 1, 3, and 5-year ROC curves are presented (**Figures S2A–C**), which indicate that our model is robust.

These findings demonstrate that Cu_riskScore is a reliable prognostic biomarker for predicting the three and five-year survival status of patients with COAD. Heatmaps (**Figure 2D**) depict the six gene expression patterns with increasing Cu_riskScore levels. Moreover, to validate these markers at the protein level, we applied immunohistochemical data from a public database (https://www.proteinatlas.org/) and found strong RNF113A, STC2 and ANKS4B in Colon Cancer samples (**Figure S2D**).

### Clinical and biological value of cuproplaisa riskScore

**Figure 3:**
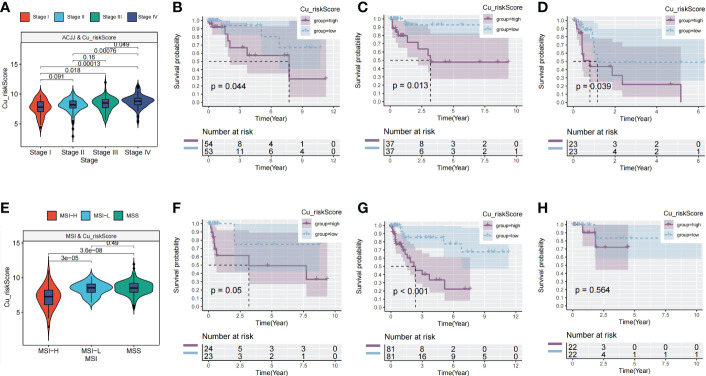
Clinical value of Cu_riskScore. **(A)** Cu_riskScore levels in four different stages in TCGA. **(B–D)** Kaplan-Meier survival analysis of the Cu_riskScore high group and Cu_riskScore low group in stage II, stage III, and stage IV, respectively. **(E)** Cu_riskScore levels in three different MSI types. **(F–H)** Kaplan-Meier survival analysis of the Cu_riskScore high group and Cu_riskScore low group in MSI-L, MSS, and MSI-H, respectively.

The AJCC stage also indicated a significant difference in Cu_riskScore, and there were significant differences between patients in stage IV and other AJCC stages (**Figure 3A**). Furthermore, in each stage (stage II, stage III, and stage IV), patients in Low and High Cu_riskScore groups exhibited significant differences (**Figures 3B–D**). Additionally, tumors belonging to the MSI-H subtype had a relatively lower Cu_riskScore than those with low microsatellite instability (MSI-L) and MSS tumors (**Figure 3E**). Moreover, in the MSS subtype, patients with a high Cu_riskScore exhibited a significantly worse survival than the Cu_riskScore low group, while this trend was not observed in the MSI-H and MSI-L groups (**Figures 3F–H**).

**Figure 4:**
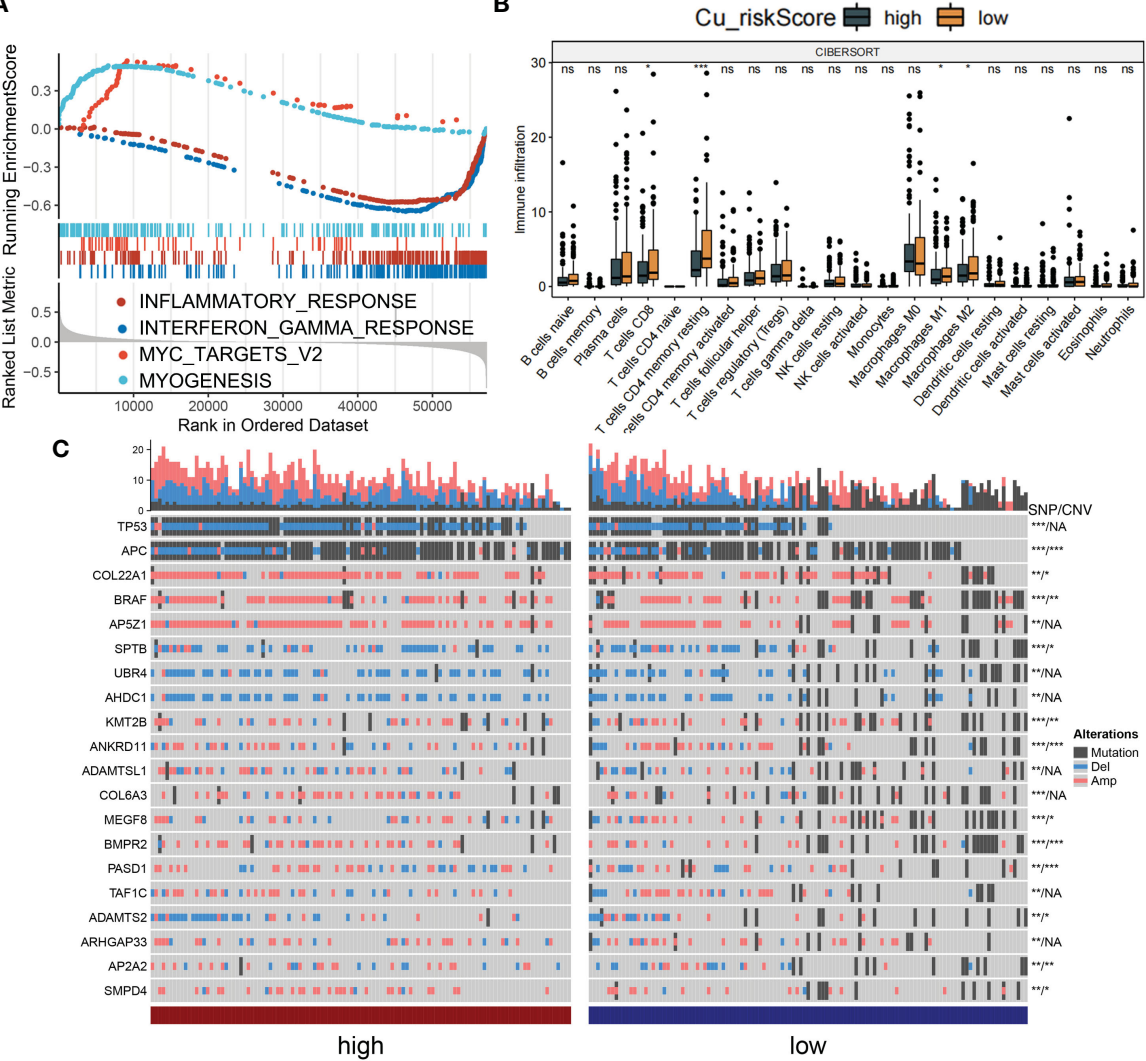
Biological value of Cu_riskScore. **(A)** GSEA running plot showing the top enriched biological process of Hallmark in TCGA. **(B)** Box plots showing immune infiltration levels in Cu_riskScore high and low based on CIBERSORTx algorithm. *P < 0.05, **P < 0.01, ***P < 0.001. ns, not statistically significant. **(C)** Heatmap showing the top 20 SNP with highest mutation rate in Cu_riskScore high and low groups and the corresponding CNV of each gene, *P < 0.05,**P < 0.01, ***P < 0.001. NA, not statistically significant.

We aimed to gain a deeper understanding of the relationship between Cu_riskScore and key biological processes, particularly in light of the remarkable performance of the Cu_riskScore on the clinical outcomes of COAD patients. GSEA was performed to determine the most highly enriched gene set in the Hallmark gene sets. The top enriched gene sets with adjusted p-value <0.1 in Hallmark are presented (**Figure 4A**). The findings revealed that, Cu_riskScore was positively related to MYOGENESIS and MYC TARGETS. The origin of SMC (smooth muscle cells) is usually of mesodermal origin after the creation of muscle cells in the process of myogenesis. SMC is critical in forming ECM (Extracellular matrix), which plays an important role in affecting the development of Colon cancer and resistance to chemotherapy. Therefore, we speculated that cuproplasia can regulate the remodeling of ECM through myogenesis. c-myc is a family of regulator genes that code for transcription factors. In cancer, MYC often leads to the increased expression of many genes, some of which are involved in cell proliferation, contributing to cancer formation (20). Thus, the genes composing Cu risk score are likely to induce poor survival by targeting MYC pathways.

Due to the strong association between immune and inflammatory responses and the Cu_riskScore, we analyzed the immune infiltration pattern of the Cu_riskScore phenotypes. QuanTIseq and xCell algorithms exhibited a higher Treg and CD8^+^ T cell infiltration in Cu_riskScore low group than high group (**Figure 4B** and **Figures S3A–C**). Low stromal and microenvironment scores were found in Cu_riskScore high group through xCell, which helped us evaluate the Tumor microenvironment status. The concordant increase in CD8+ T cells and Tregs can be attributed to enhanced immune infiltration. However, when comparing the CD8+ T cells and Tregs ratio, the high Cu_riskScore group demonstrated a lower ratio, suggesting a more immunosuppressive microenvironment (**Figure S3D**).

We then compared the genomic traits between Cu_riskScore high and low groups. We found genes such as APC, COL22A1, BRAF and KMT2B have significant SNP (single nucleotide polymorphism) and CNV (copy number variation) differences between high and low groups. While genes such as TP53, AP5Z1, UBR4 and AHDC1 only contain SNP differences (**Figure 4C**). Additionally, we attempted to focus CNV on the chromosome arm level, and we discovered that the CNV status on 1Q,5C,2Q and 3C is distinct between these two groups (**Figure S4**).

**Figure 5:**
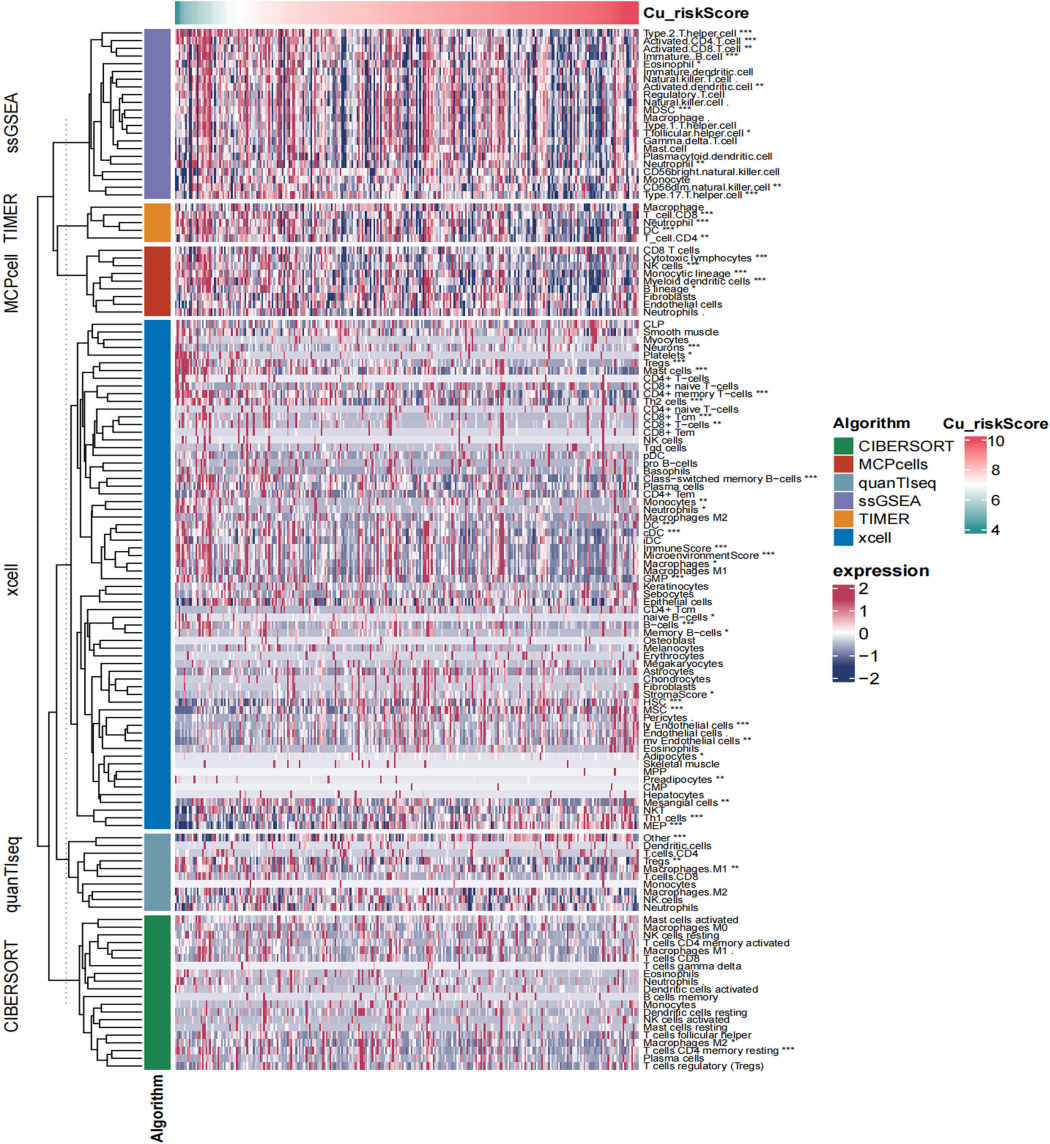
Heatmap showing the landscape of immune infiltration based on Cu_riskScore. *P < 0.05, **P < 0.01, ***P < 0.001.

A higher Cu_riskScore revealed a scarce tumor microenvironment with a significantly low stromal and microenvironment score (**Figure 5**). However, endothelial cells were found to be more abundant in the Cu_riskScore high group, indicating a higher tumor burden (**Figure 5**).

### Cuproplasia riskScore for predicting immunotherapy benefits

**Figure 6:**
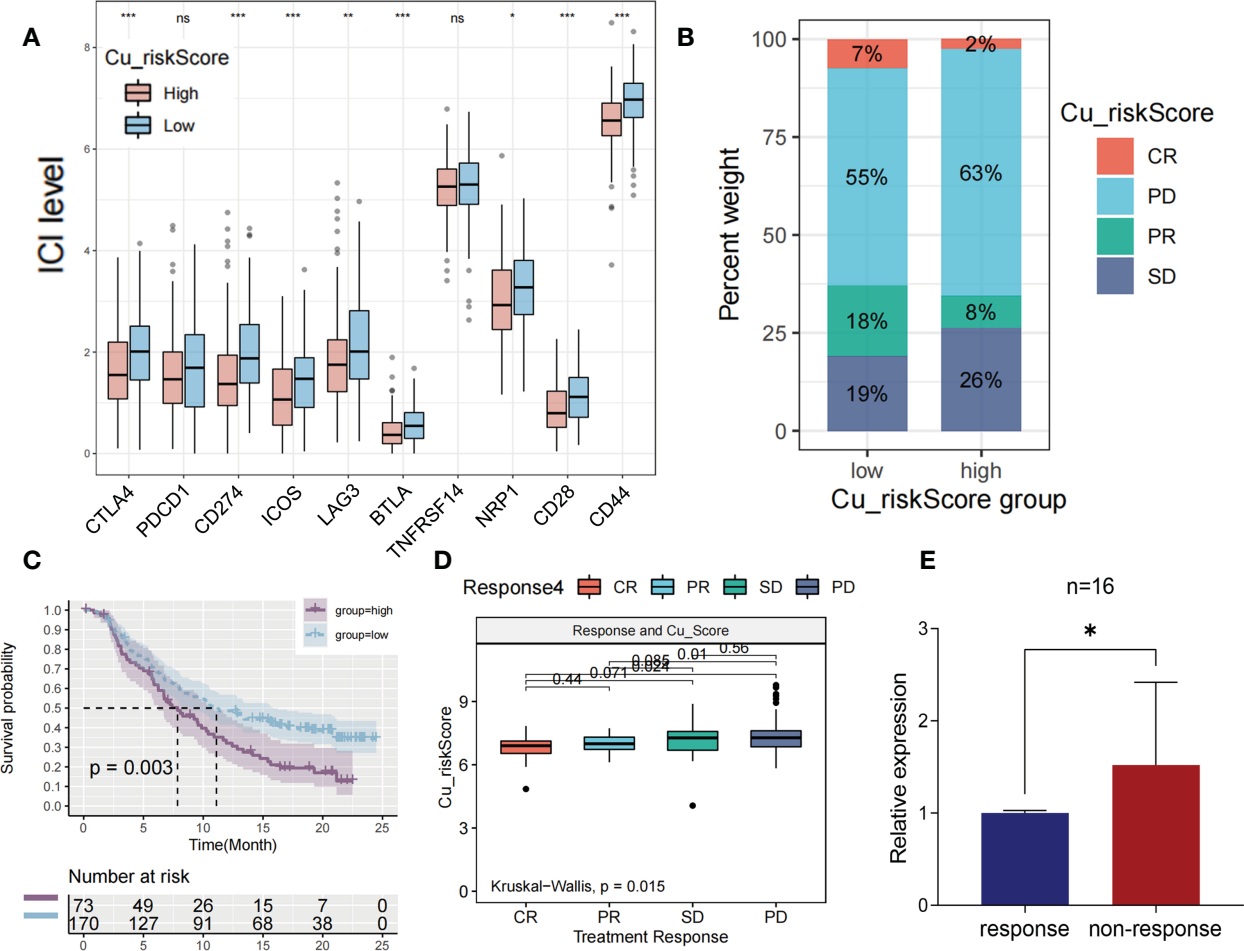
The prediction of immunotherapeutic efficacy based on cuproplasia signature **(A)** Boxplot showing the differential expression of immune checkpoint between Cu_riskScore high and low groups. **(B)** Stack bar chart showing the distribution of 4 immunotherapeutic responses in two Cu_riskScore groups in TCGA. **(C)** Kaplan-Meier survival analysis of the patients with or without immunotherapy in Cu_riskScore high and low groups. **(D)** Boxplot exhibiting the different levels of Cu_riskScore among different immunotherapeutic response groups. **(E)** Boxplot showing the level of RNF113A in 16 pairs of patients with response or non-response.

Immune checkpoint blockade (ICB) is a promising drug that blocks checkpoint proteins, such as PD-1, CTLA-4, and ICOS, from binding with their partner proteins. ICBs have emerged as anticancer treatments with unprecedented and synergistic survival benefits (21). Thus, we explored the predictive and prognostic value of the Cu_riskScore model for immune checkpoint blockade. A high Cu_riskScore was significantly associated with several immune checkpoint molecules, including PD-1, PD-L1, ICOS, CTLA4, and LAG3 (**Figure 6A**).

We then obtained samples and their clinical data from the IMvigor210 cohort to evaluate the prognostic value of the Cu_riskScore for the therapeutic effects of ICB. Patients were stratified according to their immunotherapeutic responses, including complete response (CR), progressive disease (PD), partial response (PR), and stable disease (SD). As a result, patients in Cu_riskScore high group had a higher percentage of SD and PD patients (**Figure 6B**). Moreover, high and low Cu risk scores succeeded in stratifying patients according to survival probability (**Figure 6C**). We found that patients acquiring SD and PD had significantly higher Cu_riskScore than their counterparts in the CR or PR group, indicating that high Cu_riskScore results in ICB resistance in patients with COAD (**Figure 6D**). We also performed ROC analysis to test the predictive value of Cu_riskScore (**Figure S5**). As a result, the AUC of the ROC curve is 0.586. When we used the cutoff value 7.462, to separate patients into high and low risk group, the negative predictive value (NPV) and positive predictive value (PPV) of our model are 0.72368 and 0.46707 respectively.

Based on these results, we further analyzed the difference in efficacy between PD-1 inhibitor and CTLA4 inhibitor in patients with different Cu_riskScore according to the sensitivity data of immunotherapy in the TCIA database (**Figure S6**). The results demonstrated that patients in the low Cu_riskScore group were more sensitive to CTLA4 inhibitors (P = 0.042, **Figure S6B**) and CTLA4 inhibitors in combination with PD-1 inhibitors (P = 0.025, **Figure S6D**). Taken together, these results indicate that the Cu_riskScore is likely to be associated with immunotherapeutic response and may have implications for the selection of sensitive regimens in clinical practice.

To validate our results in real world, a cohort with 32 MSI patients with colorectal liver metastases was obtained from October 17, 2022, to January 19, 2022. Since RNF113A has the foremost predictive significance, we performed qRT-PCR to assess RNF113A expressions in each sample (**Supplementary Table S5**). Patients with response to immunotherapy exhibited significantly low levels of RNF113A compared to those with non-response (**Figure 6E**), implying that RNF113A has a marked effect in predicting immunotherapy response.

## Discussion

Increasing research connects copper signaling to cell proliferation as well as tumor growth and metastasis in cancer; therefore, more studies are required to establish a link between cuproplasia-related targets and pathways to a patient’s clinical information and biological processes. Some approaches have been applied to assess patients’ copper status, such as measuring cuproenzyme activity and detecting routine blood counts because copper depletion is reflected in a decreased count of white blood cells (3). However, these methods cannot comprehensively reflect the status of cuproplasia in each sample.

To date, an increasing number of studies have focused on cuproptosis, a new form of programmed cell death caused by excess intracellular copper (22, 23). However, few studies have focused on Cu-induced proliferation, which is an important process in tumor growth. In the present study, we used WGCNA to assess the overall cuprotosis characterization by evaluating cuprotosis-related genes. Through LASSO regression, we shortened the gene list to facilitate cuprotosis pattern analysis and generated the Cu_riskScore. We then used the Cu_riskScore to depict the comprehensive landscape of clinical traits of Cuprotosis in COAD. In addition, the Cu_riskScore was used to predict long-term survival and immunotherapy benefits in BC. In general, Cu_riskScore is a decisive risk factor for different COAD subtypes.

The findings from TCGA were validated using two GEO cohorts. In the IMvigor210 cohort, immunotherapy exhibited inferior effects in those with a high Cu_riskScore. Previous findings have displayed that copper metabolism substantially influences immunotherapeutic effects. In this study, the Cu_riskScore predicted survival outcomes, particularly in patients with MSS. GSEA analysis revealed positive correlations between Cu_riskScore and some biological processes, such as inflammatory and immune responses. Correspondingly, Copper has been shown to enhance PDL1 expression, whereas copper chelation promotes ubiquitin-mediated degradation of PDL1 in colon cancer DLD1 cell lines. In an immunocompetent mouse model, Th-MYCN treatment with copper-chelating drugs enhanced tumor proliferation and survival. Combining our research with these findings indicates that copper chelation therapy can potentially enhance the antitumor immune response (24).

Tregs are CD4+ CD25+ T cells with immunosuppressive effects on the human immune system. Tregs can suppress effector T cell responses as well as the activity of other immune cells, such as mast cells, dendritic cells, and B cells, while in malignant tumors, they promote tumor progression by suppressing anti-tumor immunity (25). In this study, we used six algorithms to assess immune cell infiltration. Interestingly, the two algorithms exhibited higher Treg, CD8^+^ T cell infiltration and CD8^+^/Treg ratio in the Cu_riskScore low group than in the Cu_riskScore high group. Low stromal and microenvironment scores were found in the Cu_riskScore high group through xCell, which helped us evaluate the Tumor microenvironment status.

In conclusion, we identified a cuproplasia-related gene expression signature (Cu_riskScore) consisting of six genes and comprehensively studied the landscape of the clinical and biological characterization of this model in Colon Cancer. A higher Cu_riskScore was closely correlated with the clinical stage, constitution of TME cells, immune response, and MSS subtypes. Moreover, the Cu_riskScore was established to be a robust prognostic indicator and predictive factor for the benefits of immunotherapy. The establishment of the Cu_riskScore will assist oncologists in predicting the survival of colon cancer patients, inform the application of suitable immunotherapy, and form the basis for developing innovative therapeutic approaches.

This study had some limitations. One important drawback is the absence of external real-world RNA-seq data that might be used to corroborate and verify our findings. Another limitation was that in-depth mechanisms, such as regulation of the processes of immune response and chemokine signaling pathways, were undetermined, and further tests should be performed to confirm these findings. Moreover, single-cell sequencing should be performed to assess the relationship between cuproplasia and alteration of the TME.

## Data availability statement

The original contributions presented in the study are included in the article/**Supplementary Material**. Further inquiries can be directed to the corresponding author.

## Ethics statement

Written informed consent was obtained from the individual(s) for the publication of any potentially identifiable images or data included in this article.

## Author contributions

BZ conceived the project. YL wrote the manuscript. LS was responsible for statistical tests and data collection. HX and SW contributed to data analysis and interpretation. BC revised the manuscript and communicated with the journal and editorial office. All authors contributed to the article and approved the submitted version.
