# Supplementary figures and images for: Cuproplasia characterization in colon cancer assists to predict prognosis and immunotherapeutic response
Source: Front Oncol. 2023 Mar 16;13:1061084. doi: 10.3389/fonc.2023.1061084 (PMC10060792; doi:10.3389/fonc.2023.1061084)

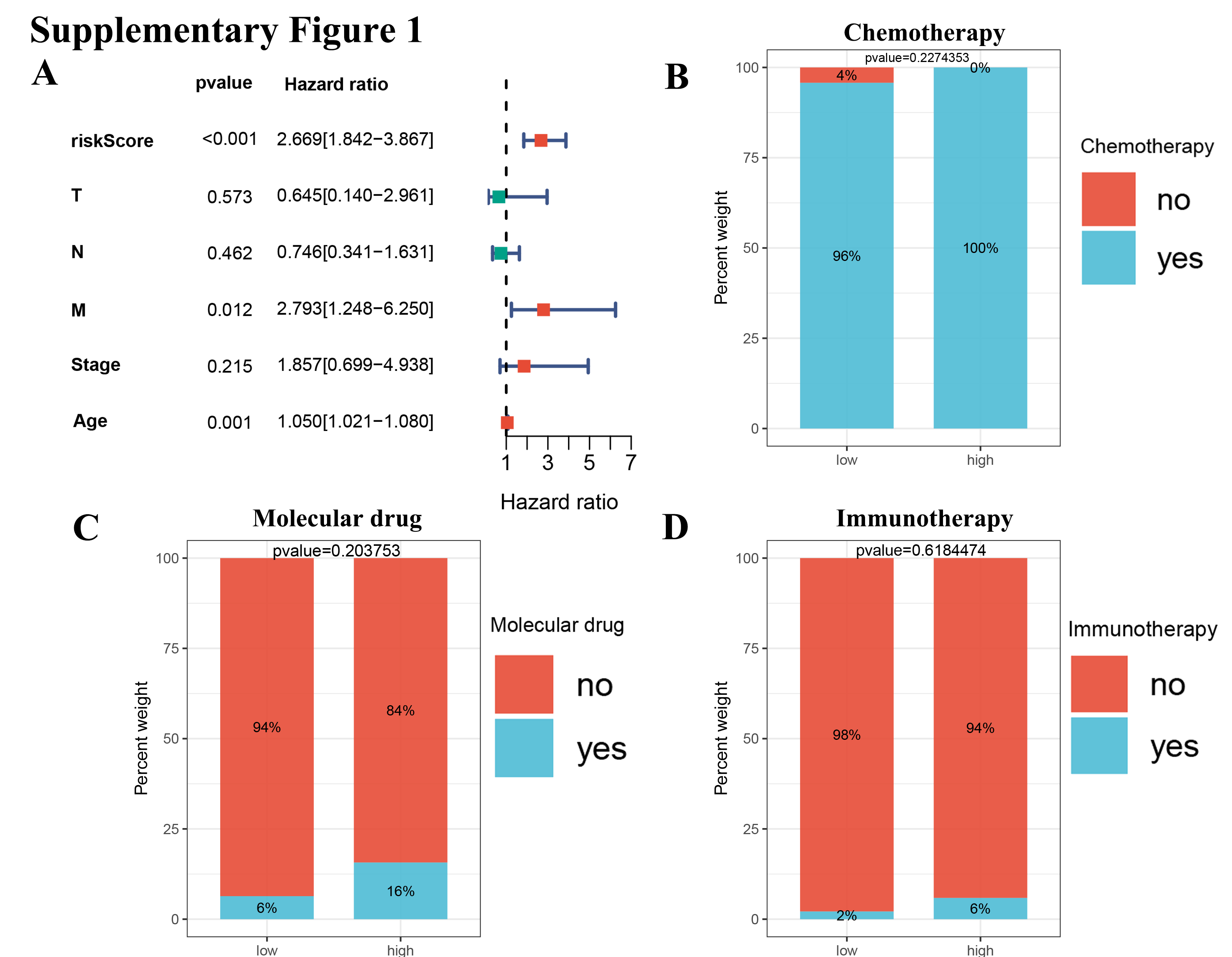

Supplement: Supplementary file 2 [file Image_1.tif]

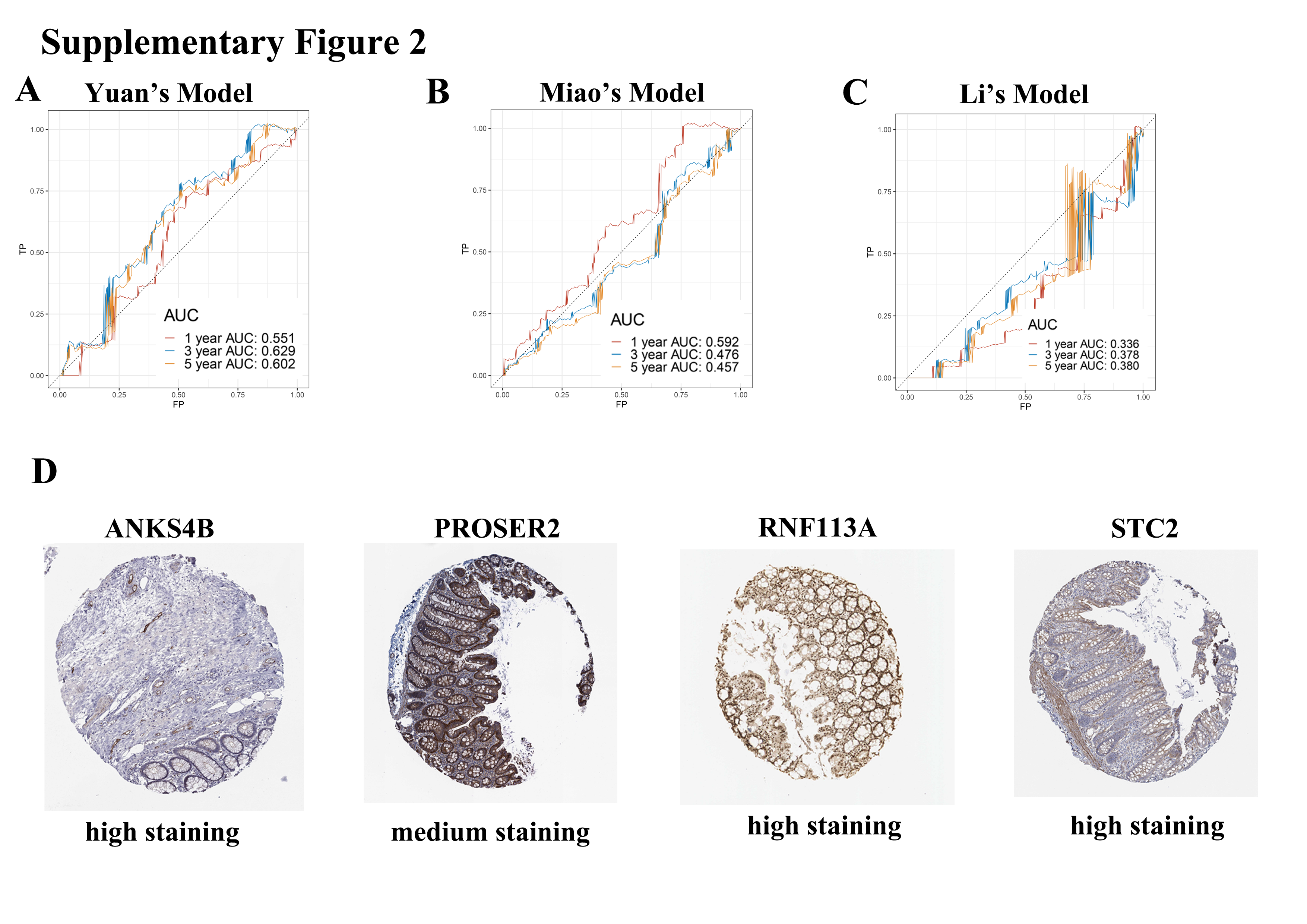

Supplement: Supplementary file 3 [file Image_2.tif]

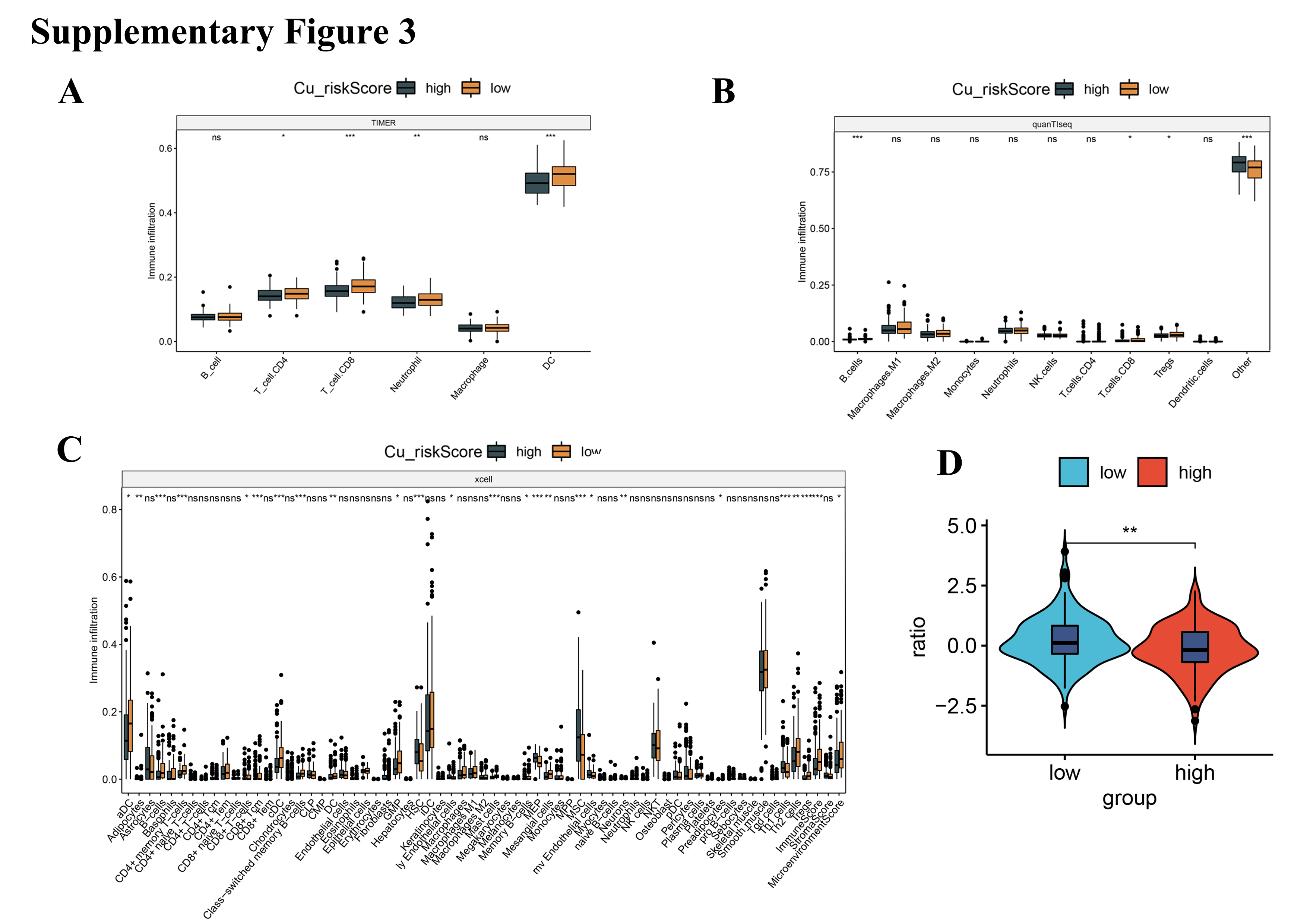

Supplement: Supplementary file 4 [file Image_3.tif]

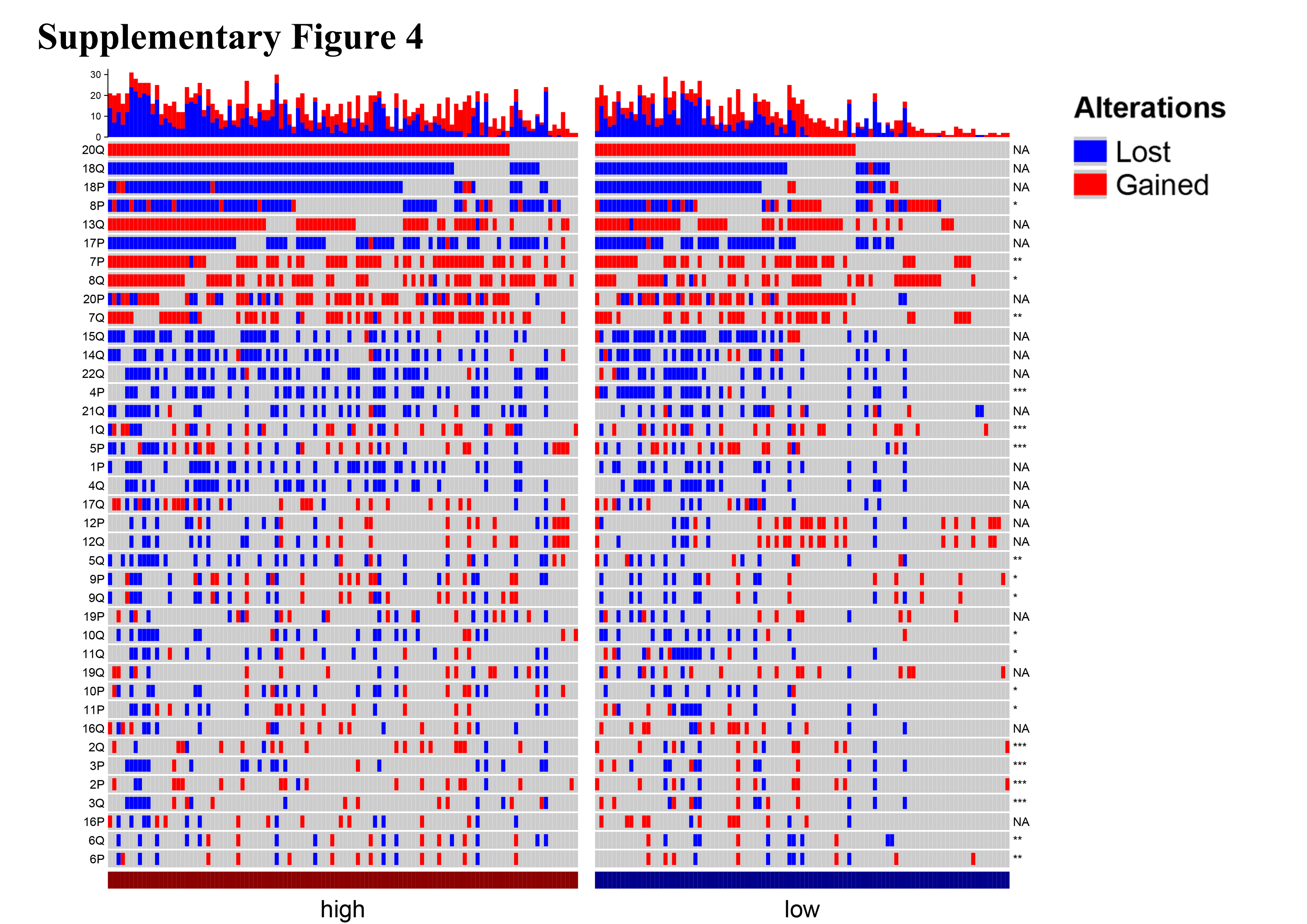

Supplement: Supplementary file 5 [file Image_4.tif]

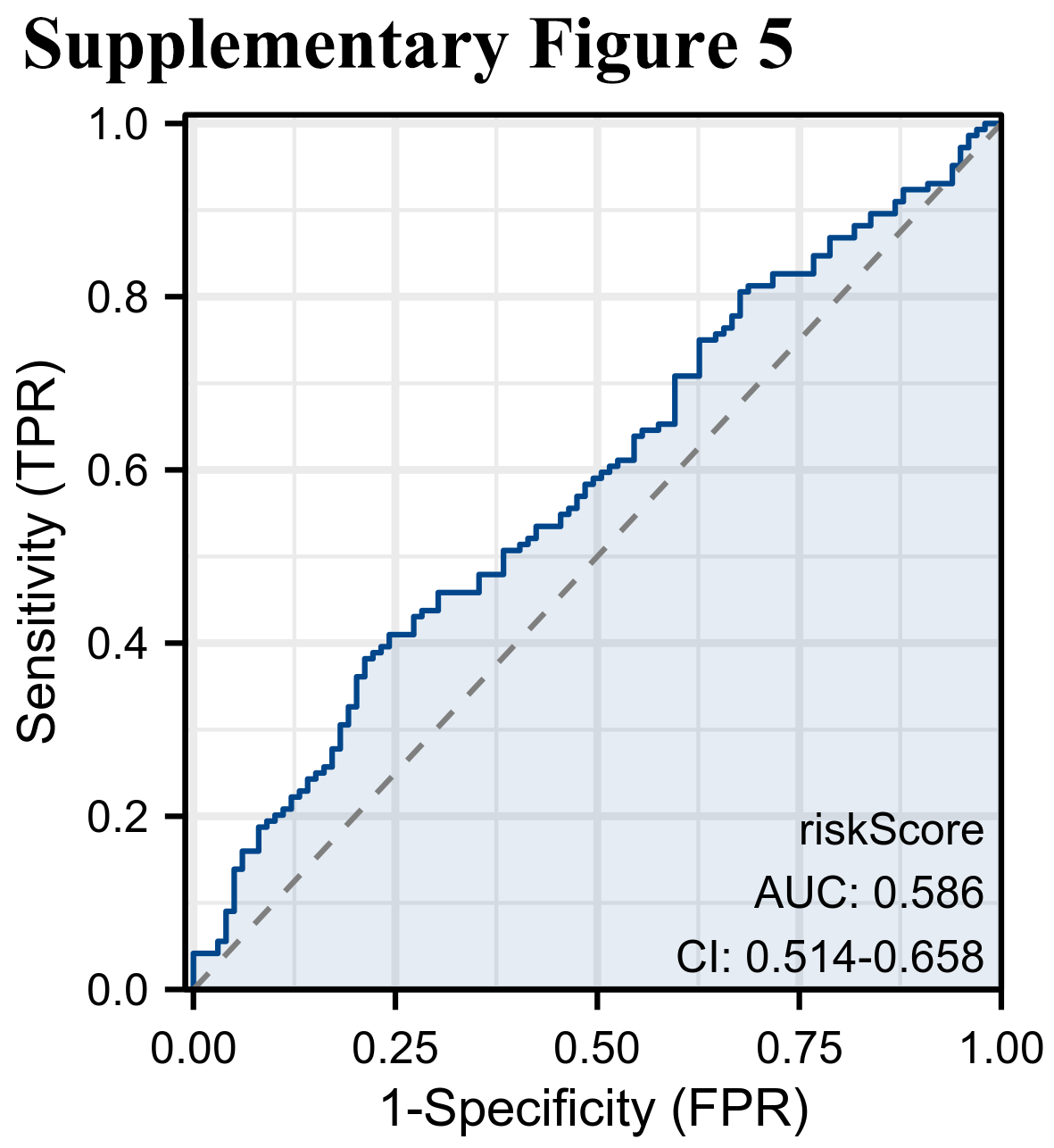

Supplement: Supplementary file 6 [file Image_5.tiff]

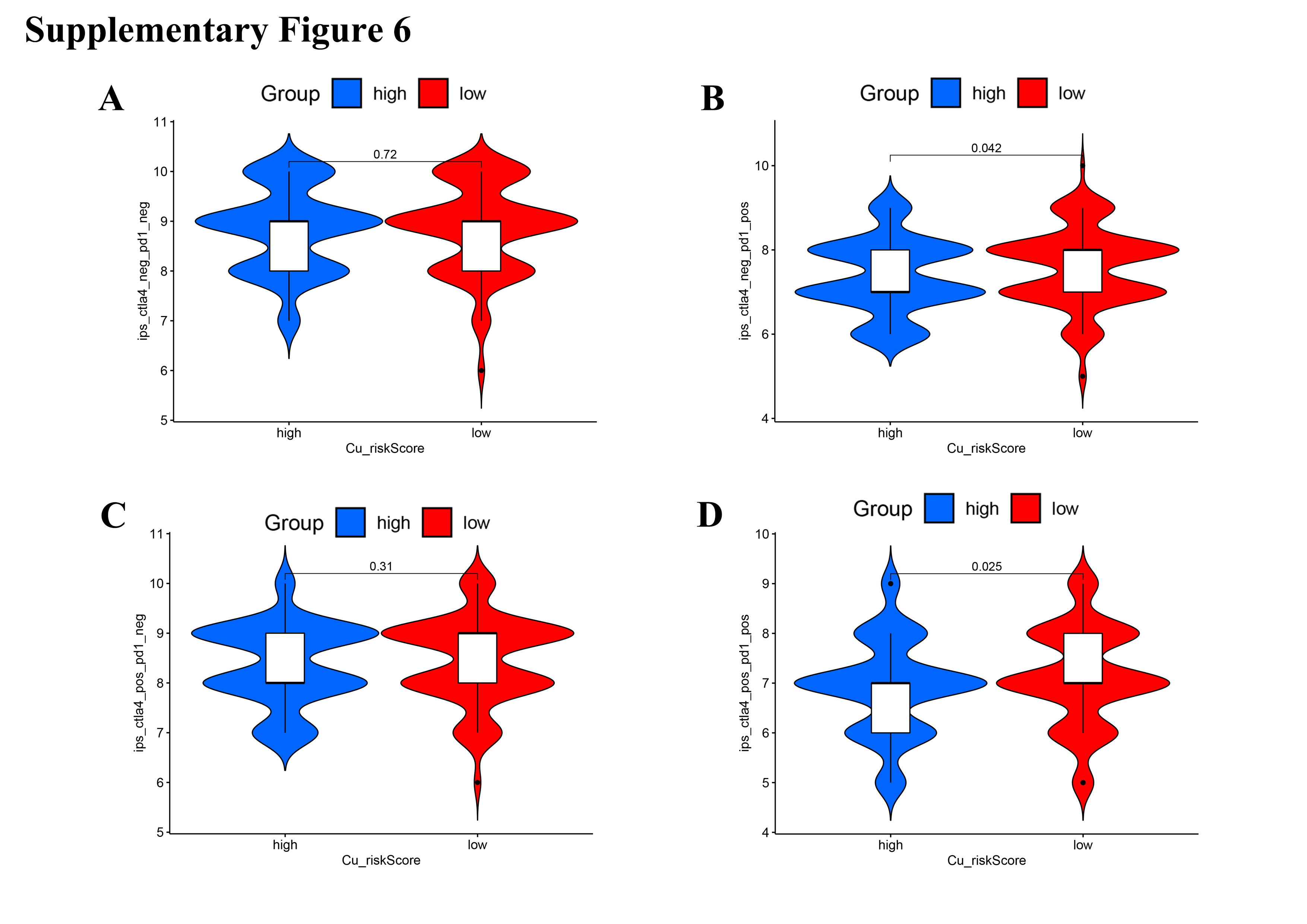

Supplement: Supplementary file 7 [file Image_6.tif]
